# ATF4 May Be Essential for Adaption of the Ocular Lens to Its Avascular Environment

**DOI:** 10.3390/cells12222636

**Published:** 2023-11-16

**Authors:** Jiawen Xiang, Anthony J. Pompetti, Adam P. Faranda, Yan Wang, Samuel G. Novo, David Wan-Cheng Li, Melinda K. Duncan

**Affiliations:** 1 Department of Biological Sciences, University of Delaware, Newark, DE 19716, USA; 2 The State Key Laboratory of Ophthalmology, Zhongshan Ophthalmic Center, Sun Yat-sen University, Guangzhou 510230, China

**Keywords:** ATF4, CREB-2, lens development, glutathione, amino acids, transporter

## Abstract

**Simple Summary:**

ATF4 is a transcription factor essential for the survival of the avascular lens due to its ability to regulate the expression of genes important for nutrient homeostasis.

**Abstract:**

The late embryonic mouse lens requires the transcription factor ATF4 for its survival although the underlying mechanisms were unknown. Here, RNAseq analysis revealed that E16.5 *Atf4* null mouse lenses downregulate the mRNA levels of lens epithelial markers as well as known markers of late lens fiber cell differentiation. However, a comparison of this list of differentially expressed genes (DEGs) with other known transcriptional regulators of lens development indicated that ATF4 expression is not directly controlled by the previously described lens gene regulatory network. Pathway analysis revealed that the *Atf4* DEG list was enriched in numerous genes involved in nutrient transport, amino acid biosynthesis, and tRNA charging. These changes in gene expression likely result in the observed reductions in lens free amino acid and glutathione levels, which would result in the observed low levels of extractable lens protein, finally leading to perinatal lens disintegration. These data demonstrate that ATF4, via its function in the integrated stress response, is likely to play a crucial role in mediating the adaption of the lens to the avascularity needed to maintain lens transparency.

## 1. Introduction

The mammalian lens is a transparent avascular tissue with a high refractive index that is required for high-resolution vision [1]. This tissue arises from mutually inductive interactions between the head ectoderm and optic vesicle that lead to lens placode induction followed by its invagination to form the lens pit, then the lens vesicle. Cells in the posterior lens vesicle then elongate to produce primary fiber cells, while the anterior cells remain proliferative and generate secondary lens fiber cells through the remainder of the organism’s life span [2]. The ability of the lens to refract light relies on numerous structural and biochemical adaptations including the precise ordering and packing of lens fiber cells [3], the accumulation of high concentrations of crystallin proteins in the lens fiber cell cytoplasm [4,5], the dismantling of light-scattering cell nuclei and organelles as lens fibers complete their differentiation [6,7], and the ability of the lens to survive and grow in the hypoxic and nutrient-poor environment arising from its avascularity [8].

The regulation of early lens development has been intensely studied for decades and key roles for numerous transcription factors including PAX6 [9,10,11], PROX1 [12,13], cMAF [14,15], and FOXE3 [16,17] have been elucidated and placed into a gene regulatory network that controls the development of the early lens from the head ectoderm [2,18]. However, much less is known about the transcriptional regulation of later steps in lens development, including the shifts in the lens fiber cell transcriptome that occur as these cells terminally differentiate [19], and the differences in gene expression profiles seen in lens fibers born at different times during development [20,21,22].

Activating transcription factor 4 (ATF4/Creb2) was first identified by its ability to specifically bind to ATF, CRE, and Tax-responsive enhancer elements in lambda-based genetic screens [23] and was later found to play important roles in mediating diverse cellular stress responses [24,25,26,27]. Mice lacking the *Atf4* gene exhibit reduced perinatal survival due to severe fetal anemia, which arises from reduced fetal liver hematopoiesis [28] while surviving mice exhibit normal adult erythropoiesis although they are infertile with delayed/improper hair growth. Notably though, adult homozygous *Atf4* null mice invariably exhibit severe microphthalmia that arises late in embryonic development due to the degeneration of the lens via P53-mediated apoptosis [29,30]. While this phenotype was first described nearly 25 years ago, the mechanisms by which ATF4 regulates lens cell survival remained a mystery. Here we re-evaluate the phenotype of the *Atf4* null lens and utilize RNA-sequencing and bioinformatics to reveal the molecular mechanisms by which ATF4 regulates the latter stages of fetal lens development.

## 2. Materials and Methods

### 2.1. Animals

All mice were maintained under pathogen-free conditions at the University of Delaware animal facility under a 14 h light/10 h dark cycle. Animals of both sexes were used in these experiments, and no sex-dependent effects were noted, consistent with our prior report [31].

Mice heterozygous for a null mutation in the *Atf4* gene [28] were obtained from Timothy Townes Lab (B6;129-*Atf4^tm1Tow^*) at the University of Alabama at Birmingham on a mixed C57Bl/6/129 genetic background. Note that the colony was not explicitly genotyped for the *Bfsp2* deletion mutation found in the 129 mouse strain [32], so it is possible that the *Atf4* null phenotype reported here is influenced by this naturally occurring mutation although RNAseq analysis suggests that *Bfsp2* mRNA is present in the lenses of these mice. *Atf4* null (ATF4^−/−^) and wildtype littermate embryos were generated by the mating of mice heterozygous for this allele, with the date that the copulatory plug was identified defined as embryonic day(E) 0.5. All genotyping was performed by Transnetyx (Cordova, TN, USA) using probes *Atf4* wt-1 and neomycin.

### 2.2. Lens Diameter Measurement

Lenses were dissected from the eye and immersed in 1× PBS. Bright-field images were taken with a Zeiss Stemi SV 11 Apo Microscope, and lens diameters were determined using ImageJ (1.53k, Wayne Rasband and contributors, National Institutes of Health, USA, http://imagej.nih.gov/ij, Java 1.8.0_172 (64-bit). Average lens diameters were calculated and differences were assessed using the F test and two-tailed Student *t*-test. Data are presented as mean ± SEM, with differences considered significant at *p* ≤ 0.05.

### 2.3. Histological Evaluation

Embryos were dissected and heads fixed in Modified Davidson’s Fixative (Electron Microscopy Sciences, Hatfield, PA, USA, 64133-50) at room temperature overnight, then transferred into 70% ethanol. Fixed samples were paraffin-embedded at the University of Delaware Comparative Anatomy Laboratory and 5 μm sections were stained with hematoxylin and eosin using standard methods.

### 2.4. Immunostaining

Fresh tissue from embryonic mice was embedded in OCT (Tissue-Tek Sakura, Torrance, CA, USA, 4583) and 14 μm cryosections were prepared as previously described [33]. Sections were fixed under conditions specific to each antibody (Supplemental Table S6) and washed 3 times in PBS for 10 min each. Sections were blocked using a solution of 5% normal goat serum (NGS, Jackson ImmunoResearch, West Grove, PA, USA, 005-000-121), 2% bovine serum albumin (BSA, Sigma-Aldrich, St Louis, MO, USA, A2153), and 0.2% Triton X-100 (Sigma-Aldrich, St Louis, MO, USA, T8787) in 1× PBS for 1 h, followed by three 10 min 1× PBS washes. The primary antibody was diluted in blocking buffer as described in Supplemental Table S6, overlaid on the sections, and incubated at 4 °C overnight. Following three 10 min washes with 1× PBS, slides were incubated in secondary antibody solution (5%NGS, 2% BSA, Draq5 (1:2000), 1:200 dilution of appropriate Alexa Fluor-labeled secondary antibody (Thermofisher, Waltham, MA, USA) in 1× PBS with or without the addition of fluorescein-labeled alpha-smooth muscle actin antibody) at room temperature for 1 h. Following three 10 min washes with PBS, slides were coverslipped and imaged on an LSM 780 Zeiss confocal microscope. Immunofluorescence was quantified by measuring mean fluorescence intensity (MFI) in the region of interest using Image J as previously described [34]. The number of animals analyzed in each experiment (biological replicates) is noted in the figure legends (i.e., “*n*”). Data were assessed using an F test and two-tailed Student *t*-test on data generated from a minimum of three biological replicates. Data are presented as mean ± SEM, and differences were considered significant at *p* ≤ 0.05. The confocal imaging of all slides in the same experiment was imaged the same day using the same imaging parameters to allow direct comparison of experimental conditions and reduce variability. While quantification was performed on native images to ensure that signals were in the linear range, in some cases, brightness and contrast were adjusted post-confocal acquisition to create the figures presented here for optimum viewing, but in all cases, identical adjustments were made to images that are intended to be directly compared.

### 2.5. RNA Sequencing and Data Analysis

Lenses were dissected from E16.5 embryos generated from *Atf4*^+/−^ intercross matings, and frozen on dry ice, while the embryos were genotyped as above. RNA was harvested from single pairs of wildtype and *Atf4* null lenses using the RNeasy Mini Kit from Qiagen (Germantown, MD, USA, catalog 74104) and shipped to DNAlink USA (Los Angeles, CA, USA) for library preparation and RNAseq analysis. RNA libraries were prepared using the SMARTer Stranded Total RNA-Seq Kit-Pico Input Mammalian (Takara Bio, Inc., Tokyo, Japan) and sequenced on a NovaSeq 6000 (Illumina, San Diego, CA, USA). The raw fastq files were run through Trim Galore [35] to remove library adapters and low-quality/short reads. Trimmed reads were then aligned to the RN45S rRNA sequence that encodes 28S, 18S, and 5S ribosomal RNAs with HISAT2, and any aligned reads were removed, then the resulting file was processed to create trimmed fastq files suitable for downstream analyses.

The *Mus musculus* GRCm39 Ensembl Release 104 primary assembly gene transfer format (GTF) file was obtained from https://ftp.ensembl.org/pub/release-104/fasta/mus_musculus/dna/ (accessed on 11 June 2021) and splice sites/exons were extracted. A comprehensive annotation table was then constructed by combining a table containing these features [36] with GRCm39 genome annotations obtained from Biomart [37,38]. Trimmed fastq files were aligned to the GRCm39 annotation file and each read assigned to a gene by HTSeq [39], and count files for each aligned gene generated. These primary count files were fed into EdgeR [40,41,42], where TMM normalization was performed and dispersion was estimated using a weighted likelihood empirical Bayes approach. The log2 fold change for each gene was calculated with the exactTest function in edgeR that uses gene-wise exact tests and data were expressed so that positive fold changes represent elevated expression in the Atf4 null lens compared to wildtype littermate controls. The normalized abundance of each transcript in each sample was calculated as fragments per kilobase million (FPKM).

Differentially expressed genes (DEGs) are those whose expression differences between wildtype and control exhibited false discovery rate (FDR)-corrected p values of less than 0.05. This DEG list was then further filtered using criteria previously proposed to identify genes whose expression differences were considered likely to be “biologically significant” [43] to the lens (absolute log fold change greater than 1, an absolute change in FPKM between the two conditions tested greater than 2, and at least one condition with an FPKM greater than 2). Gene enrichment and pathway analysis were performed on the entire list of present genes considering all statistically significant DEGs between the wildtype and Atf4 null lens using Adviata’s iPathway guide package (Ann Arbor, MI, USA).

Cross-experiment comparisons (Table 1) were used to predict whether ATF4 is involved in known biological processes by comparing the overlap between the DEGs detected in the *Atf4* null lens and those detected in other experiments of interest. First, a matrix is created that compares the proportions of non-differentially expressed genes to differentially expressed genes between the two experiments, and a one-sided Fisher’s Exact test would compare the proportions and test for enrichment. The second was to look at the proportion of differentially expressed genes between the experiments that were up-regulated and down-regulated. The top-left quadrant would indicate concordant up-regulation and the bottom-right quadrant would indicate concordant down-regulation. The top-right and bottom-left quadrants would indicate discordant regulation. A two-sided Fisher’s Exact test would compare these proportions and look at whether genes are observed going in a direction more than expected at random.

### 2.6. Lens Protein Determination

Lenses were collected from E16.5 embryos generated from *Atf4*^+/−^ intercross matings, frozen on dry ice, and genotyped as described above. Each pair of lenses from wildtype and Atf4 null embryos was homogenized in 70 μL of RIPA buffer (protease inhibitor 1:100 added) and the supernatant was collected following centrifugation (14,000× *g*, 4 °C, 15 min). Protein concentration was determined by the Pierce BCA assay (Thermo-Fisher Scientific, Waltham, MA, USA) using kit instructions from a standard curve created from 125–2000 μg/mL bovine serum albumen. Protein per lens (μg) was calculated by the protein concentration and lens average volume derived from the average diameter. Data are presented as mean ± SEM and were statistically analyzed by F test and two-tailed Student *t*-test, with differences considered significant at *p* ≤ 0.05.

### 2.7. Glutathione Assays

E16.5 WT and *Atf4* null lenses were collected, and each pair of lenses was homogenized in 120 μL of 2 mM EDTA (in 1× PBS) buffer on ice, and the supernatant collected after centrifuging at 14,000× *g* at 4 °C for 10 min. Glutathione levels were determined following the protocol given in the Promega GSH-Glo Glutathione assay kit (Madison, WI, USA, v6911) and the luminescence signal was measured using a Promega GlowMax plate reader set on the default luminescence protocol. Standard curves were created by diluting a 5 mM standard GSH stock into 0.3125, 0.625, 1.25, 2.5, and 5 μM concentrations to calculate and sample GSH concentrations. The difference between data from WT and *Atf4* nulls was assessed via the F test and two-tailed Student *t*-test. Data are presented as mean ± SEM, and differences were considered significant at *p* ≤ 0.05.

### 2.8. Reactive Oxygen Species Assays

E16.5 lenses were collected and each pair of lenses was immersed in room temperature Medium 199 (Corning, Corning, NY, USA). Each pair of lenses was then transferred into one well of 96-well plates, with 200 μL 7.5 μM dehydrorhodamine 123 (Invitrogen, Carlsbad, CA, USA, D23806) in Media 199. Plates were placed on ice for 30 min, then washed 3 times with cold 1× PBS, and fluorescence detection was performed on a Promega GlowMax plate reader. Data from wildtype and Atf4 null lenses were statistically analyzed using F and two-tailed Student’s *t*-test. Data are presented as mean ± SEM, and differences were considered significant at *p* ≤ 0.05.

### 2.9. Free Amino Acid Detection

E16.5 WT and ATF4^−/−^ lenses were collected, and each pair of lenses was homogenized in 40 μL of 100 mM ammonium acetate, with 200 μM Ala isotope (L-Ala-1-^13^C, Sigma-Aldrich, 489867) added as an internal standard, then centrifuged at 14,000× *g* at 4 °C for 15 min. The supernatant was collected, and then 120 μL of 0.2% formic acid in acetonitrile was added to each tube. The solution was placed on ice for 1 min, vortexed for 1 min, then centrifuged at 14,000× *g* at 4 °C for 15 min, and the supernatant was used for amino acid determination. LC-MS/MS analysis was performed by the University of Delaware Mass Spectrometry Core Facility using a Q-Exactive Orbitrap interfaced with Ultimate 3000 LC system (Thermo Fisher Scientific). Chromatographic separation was achieved using an Intrada amino acid column (3 µm, 3 × 50 mm column), with mobile phase A consisting of 100 mM ammonium formate and mobile phase B consisting of acetonitrile containing 0.1% formic acid. Sample elution was performed using a gradient starting at 8% mobile phase A/92% mobile phase B for 3 min, then increasing to 70% mobile phase A/30% mobile phase B for 12 min, with a column temperature of 35 °C and a flow rate of 0.6 mL/min. Acquisition of MS data scans (50–750 *m*/*z*) was performed using a data-dependent top 5 method, dynamically selecting the most abundant precursor ions from the survey scan for high-energy collision-induced dissociation (HCD) fragmentation using a stepped normalized collision energy of 25, 30, and 35 eV. Raw LC-MS data were analyzed by Xcalibur software (Thermofisher version 4.5), and amino acid concentrations were determined by comparisons with a standard amino acid mix diluted into 0.1, 1, 2, 5, and 10 μM concentrations to create a standard curve. The amino acid amount per lens was then calculated for each sample and statistically assessed using the F test and two-tailed Student’s *t*-test. Data are presented as mean ± SEM, and differences were considered significant at *p* ≤ 0.05.

## 3. Results

**Figure 1:**
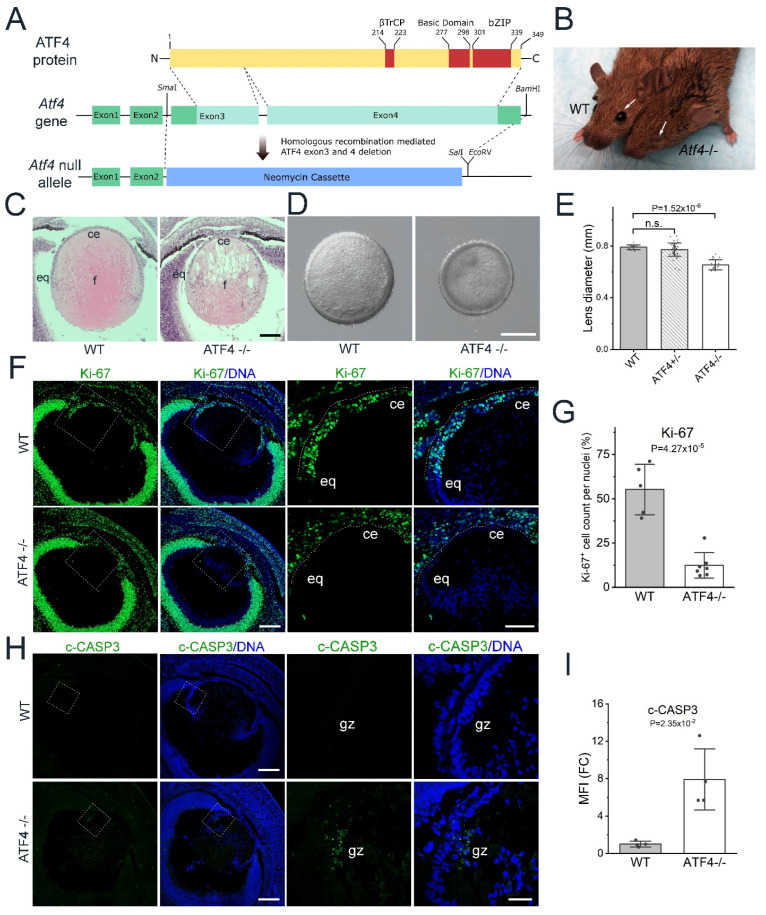
*Atf4* is required for lens epithelial cell proliferation and lens fiber cell survival during late embryonic development. (**A**) Mouse ATF4 protein primary structure (N, N terminus; C, C terminus; βTrCP, βTrCP-binding domain) and wildtype mouse *Atf4* gene structure (green represents non-coding portions of exons; aqua represents coding sequence). Note that alternate splicing can result in a three-exon transcript where exons 1,2 and the intervening intron encompass “exon 1” as described in the original paper describing this *Atf4* allele [28] with both of these splice variants producing the same protein [44]. The final structure of the *Atf4* null allele where the coding sequence located in exons 3 and 4 (exons 2 and 3 in [28]) was removed by homologous recombination (Sma1, BamH1, SalI, and EcoRV are restriction enzyme sites used in construct production) and replaced with a neomycin resistance cassette (blue). (**B**) A two-month-old wildtype mouse (left) and its *Atf4* null (−/−) littermate (right). (**C**) Hematoxylin and eosin staining of paraffin sections generated from E16.5 wildtype eye (left) and E16.5 *Atf4* null eye (right, scale bar = 100 μm). (**D**) Bright-field image of representative E16.5 lenses microdissected from wildtype (WT, left) and *Atf4* null (ATF4^−/−^; right) mice (scale bar = 300 μm). (**E**) Quantification of the diameters of lenses microdissected from E16.5 wildtype (WT, *n* = 18), heterozygote (ATF4^+/−^, *n* = 48), and homozygote (ATF4^−/−^, *n* = 23) mice. (**F**) Representative image obtained from sections of E16.5 wildtype and *Atf4* null eyes immunostained for the proliferation marker Ki-67 (green) and labeled with the DNA dye Draq5 (blue). Images on the left were generated from a 3 × 3 tile scan with a 20× objective (scale bar = 200 μm), and the boxed region is shown in a zoomed-in view on the right (scale bar = 100 μm). (**G**) Quantification of the percentage of lens epithelial cell nuclei positive for Ki-67 (WT, *n* = 5; ATF4^−/−^, *n* = 5). (**H**) Immunostaining of E16.5 wildtype and *Atf4* null lenses for the apoptotic marker cleaved caspase 3 (green) and labeled with the DNA dye Draq5 (blue). Images on the left were collected via a 3 × 3 tile scan using a 20× objective (scale bar = 200 μm) with the boxed region representing the zoomed-in view shown on the right (scale bar = 40 μm). (**I**) Quantification of the fold change (FC) in mean fluorescence intensity (MFI) of cleaved-Caspase3 staining between WT and *Atf4* null lenses (WT, *n* = 4; ATF4^−/−^, *n* = 4). *Abbreviations*: ce, central epithelium; eq, equator; gz, germinative zone; WT, wildtype.

The first studies on ATF4 function in lens development reported that *Atf4* deletion led to morphological abnormalities in the lens starting in late embryonic development, with total disintegration of the lens seen by birth due to epithelial and/or fiber cell apoptosis [29,30]. While these investigations revealed that ATF4 is important for lens development and/or its homeostasis, the mechanisms by which ATF4 regulates these processes were unknown. In order to re-evaluate these phenotypes, we obtained a different *Atf4* null allele (*Atf4^tm1Tow^*) established by replacing exon 3 and 4 of the *Atf4* gene, with a neomycin phosphotransferase cassette [28] (Figure 1A).

### 3.1. ATF4 Is Required for Lens Epithelial Cell Proliferation and Survival during Late Embryonic Development

As previously described, mice homozygous for the *Atf4^tm1Tow^* allele exhibit high perinatal lethality [28], and consistent with the prior reports on other *Atf4* null alleles, surviving adult homozygotes lack externally visible eyes [28] (Figure 1B, arrows) while the remaining eye remnant lacks lens material [29]. Hematoxylin and eosin staining revealed that *Atf4^tm1Tow^* null lenses were typically grossly normal at E14.5 (Supplemental Figure S1), but by E16.5, *Atf4^tm1Tow^* null lenses are significantly smaller than normal (Figure 1C–E) with vacuoles apparent in central lens fibers (Figure 1C).

Staining of *Atf4^tm1Tow^* null lenses for KI-67, a marker of cell proliferation [45], revealed that over 50% of wildtype E16.5 LECs were active in the cell cycle, while fewer than 20% of mutant LECs were proliferating (Figure 1F,G). As apoptosis was previously reported to drive lens loss in *Atf4* null mice, we stained E16.5 wildtype and *Atf4^tm1Tow^* null lenses for cleaved-Caspase3 (c-CASP3), an apoptosis marker in the lens [46]. While, as expected, wildtype lenses had no apparent c-CASP3 staining, the germinative zone lens epithelium of *Atf4^tm1Tow^* null lenses exhibited patchy regions of c-CASP3 positivity (Figure 1H,I). Overall, the terminal lens phenotype of *Atf4^tm1Tow^* null lenses (loss of lens material after birth) was similar to the lens phenotype of the two previously described alleles [29,30]. However, the loss of lens material from *Atf4^tm1Tow^* homozygotes appears slightly delayed compared to that reported by Hettmann et al. [29]. These data suggest that ATF4 is required for LEC proliferation and survival in the late embryonic mouse lens.

### 3.2. Unfolded Protein Response Markers Are Only Modestly Affected in the Atf4 Null Lens

**Figure 2:**
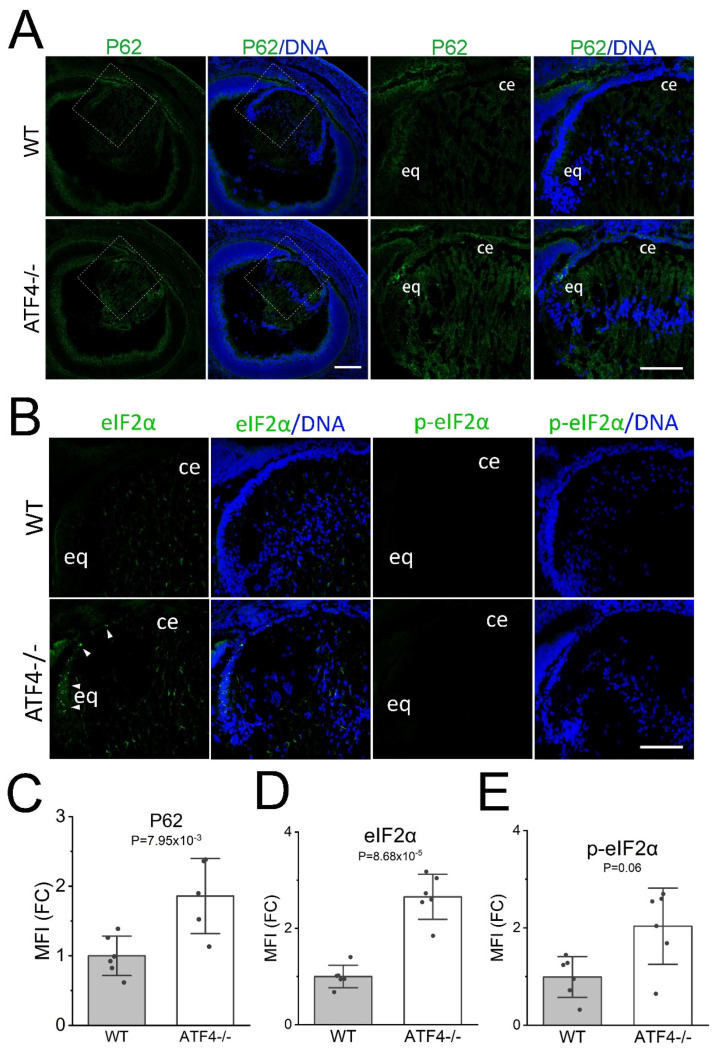
Only portions of the autophagy pathway are induced in the *Atf4* null lens. (**A**) Immunostaining of E16.5 wildtype and *Atf4* null (Atf4^−/−^) lens for P62 (green) that is encoded by the *Sqtm1* (Sequestosome-1, Sqtm1) gene counterstained with Draq5 to detect DNA (blue). Images on the left were generated from a 3 × 3 tile scan with a 20× objective (scale bar = 200 μm), and the boxed region is shown in a zoomed-in view on the right (scale bar = 100 μm). (**B**) Immunostaining of E16.5 wildtype and *Atf4* null lens for the eIF2α (left panels, green) and p-eIF2α (right panels, green) counterstained with Draq5 to detect DNA (blue) (scale bar = 100 μm). *Abbreviations*: ce, central epithelium; eq, equator. (**C**) Quantification of the mean fluorescent staining intensity of P62 immunostaining in the E16.5 wildtype (*n* = 6) and *Atf4* null (*n* = 5) lens. (**D**) Quantification of the mean fluorescent staining intensity of eIF2α immunostaining in the E16.5 wildtype (*n* = 6) and *Atf4* null (*n* = 6) lens. (**E**) Quantification of the mean fluorescent staining intensity of p-eIF2α immunostaining in the E16.5 wildtype (*n* = 6) and *Atf4* null (*n* = 6) lens.

The unfolded protein response (UPR) allows cells to calibrate the folding capacity of the endoplasmic reticulum to the flux of newly produced secreted/membrane proteins, while its chronic activation leads to cell dysfunction leading to apoptosis [47]. Notably, numerous components of the UPR pathway are modestly activated in the developing lens [48] while chronic UPR can lead to cataract [49,50,51]. As ATF4 is a known UPR regulator [52], we investigated whether E16.5 *Atf4^tm1Tow^* null lenses have a defect in UPR during lens development. BIP/*grp78* is an endoplasmic reticulum chaperone whose expression typically upregulates during UPR [53]. As previously shown, BIP levels are highest in the lens epithelium and newly elongating fiber cells in wildtype lenses [48], while they are modestly attenuated in E16.5 *Atf4^tm1Tow^* null lenses (Supplemental Figure S2A,B). Similarly, the protein levels of neither CHOP/*Ddit3*, an ATF4 target, [54] nor XBP1 whose protein is selectively produced when the IRE1 UPR signaling pathway is activated [55] were significantly altered in E16.5 *Atf4^tm1Tow^* null lenses (Supplemental Figure S2A,C,D). However, the protein levels of eIF2α, a translational regulator that selectively synthesizes UPR pathway proteins, were modestly elevated in E16.5 *Atf4^tm1Tow^* null equatorial lens epithelium (Figure 2B,D).

### 3.3. RNAseq Revealed That ATF4 Regulates a Subset of the Lens Transcriptome

Although *Atf4^tm1Tow^* null lenses exhibit modest changes in UPR response pathways, the genes that ATF4 regulates in the lens were still obscure. Thus, we performed RNAseq on lenses isolated from E16.5 *Atf4^tm1Tow^* null embryos and their wildtype littermates to gain an unbiased insight into the potential function of ATF4 in the lens. Consistent with other studies [12,56], both wildtype and *Atf4^tm1Tow^* null lenses express approximately 15,000 genes at sufficient abundance to pass the “filter by expression” threshold set in EdgeR [40,41,42]. Of these, 983 genes were differentially expressed based on FDR-corrected *p* value, with 368 genes upregulated and 615 genes downregulated (Supplemental Figure S3A, Supplemental Table S1). Filtering this list further using criteria that we previously proposed to robustly identify genes whose differential expression was likely to have biological significance (absolute fold change > 2, expression in at least one condition greater than 2 FPKM) [43] resulted in a list of 418 genes of potential interest representing 125 upregulated and 293 downregulated genes, including *Atf4* itself (Supplemental Table S1). While some *Atf4* mRNA was still detected in *Atf4^tm1Tow^* null lenses, mapping of these transcripts to the mouse genome revealed that these reads solely represented residual transcription of the non-coding exons 1 and 2 and the intervening intron that is included in some *Atf4* splice variants (Supplemental Figure S3B). The deletion of the *Atf4* coding sequences in this mutant did not affect the expression of its upstream gene, *Mief1*, although the gene immediately downstream of the *Atf4* locus, *rps19bp1* that encodes AROS, a suppressor of P53 activity [57], was 2-fold downregulated, which may drive part of the P53-mediated apoptosis seen in the lens of *Atf4* null mice [29]. These data have been deposited in the Gene Expression Omnibus under accession number GSE206760.

### 3.4. Atf4 Null Lens Epithelial Cells Downregulate Known Lens Epithelial Markers While Upregulating α-SMA Expression

**Figure 3:**
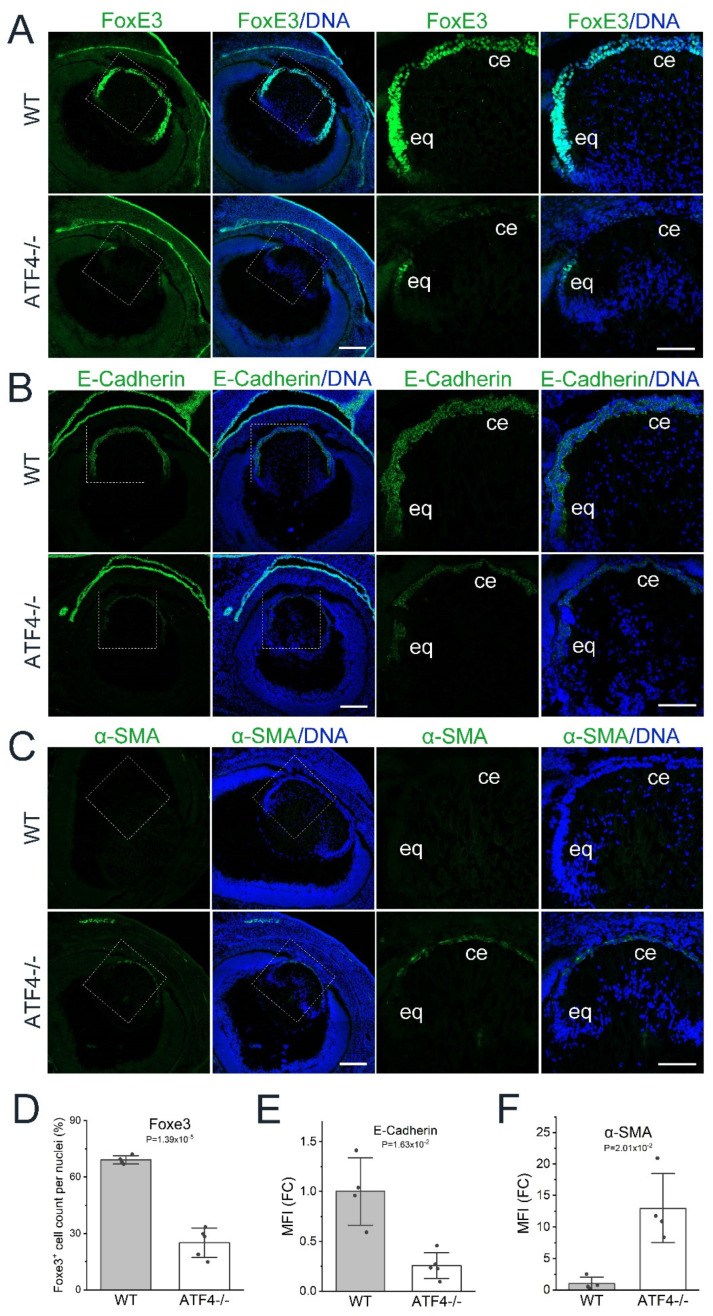
*Atf4* null lens epithelial cells downregulate known lens epithelial markers while upregulating α-SMA expression. (**A**) Immunostaining of E16.5 wildtype and *Atf4* null lens for FoxE3 (green) counterstained with Draq5 to detect DNA (blue). Images on the left were generated from a 3 × 3 tile scan with a 20× objective (scale bar = 200 μm), and the boxed region is shown in a zoomed-in view on the right (scale bar = 100 μm). (**B**) Immunostaining of E16.5 wildtype and *Atf4* null lens for E-cadherin (green) counterstained with Draq5 to detect DNA (blue). Images on the left were generated from a 3 × 3 tile scan with a 20× objective (scale bar = 200 μm), and the boxed region is shown in a zoomed-in view on the right (scale bar = 100 μm). (**C**) Immunostaining of E16.5 wildtype and *Atf4* null lens for α-SMA (green) counterstained with Draq5 to detect DNA (blue). Images on the left were generated from a 3 × 3 tile scan with a 20× objective (scale bar = 200 μm), and the boxed region is shown in a zoomed-in view on the right (scale bar = 100 μm). Abbreviations; ce, central epithelium; eq, equator. (**D**) Quantification of the percentage of lens epithelial cell nuclei positive for FoxE3 (WT, *n* = 5; ATF4^−/−^, *n* = 5). (**E**) Quantification of the fold change (FC) in mean fluorescence intensity (MFI) of E-cadherin staining between WT (*n* = 4) and Atf4 null (*n* = 5) lenses. (**F**) Quantification of the fold change (FC) in mean fluorescence intensity (MFI) of α-SMA staining between WT (*n* = 4) and *Atf4* null (*n* = 4) lenses.

Inspection of the DEG list revealed that the mRNA levels of *FoxE3* [16] and E-cadherin/*Cdh1* [58], which are known regulators of the lens epithelial phenotype, were downregulated in the *Atf4* null lens (see Table 2, Supplemental Table S1). Immunostaining for FOXE3 (Figure 3A,D) and E-cadherin (Figure 3B,E) protein revealed that these reduced mRNA levels resulted in significant reductions in their protein expression in the E16.5 *Atf4* null lens. Meanwhile, α-smooth muscle actin (α-SMA) expression, which is often considered as a diagnostic marker of lens EMT [59,60], increased in E16.5 *Atf4* null lenses at both the mRNA (Supplemental Table S1) and protein (Figure 3C,F) levels. However, the upregulation of αSMA expression in the *Atf4* null lens appears to not indicate bona fide fibrotic transformation of the lens epithelium as the mRNA levels of other classic markers of LEC fibrosis such as tenascin C, fibronectin, and collagen I [61] are not significantly differentially expressed in *Atf4* null lenses (not shown, Supplemental Table S1).

### 3.5. ATF4 Likely Regulates a Portion of the Lens-Preferred Transcriptome Including Markers of Late Fiber Cell Differentiation

**Figure 4:**
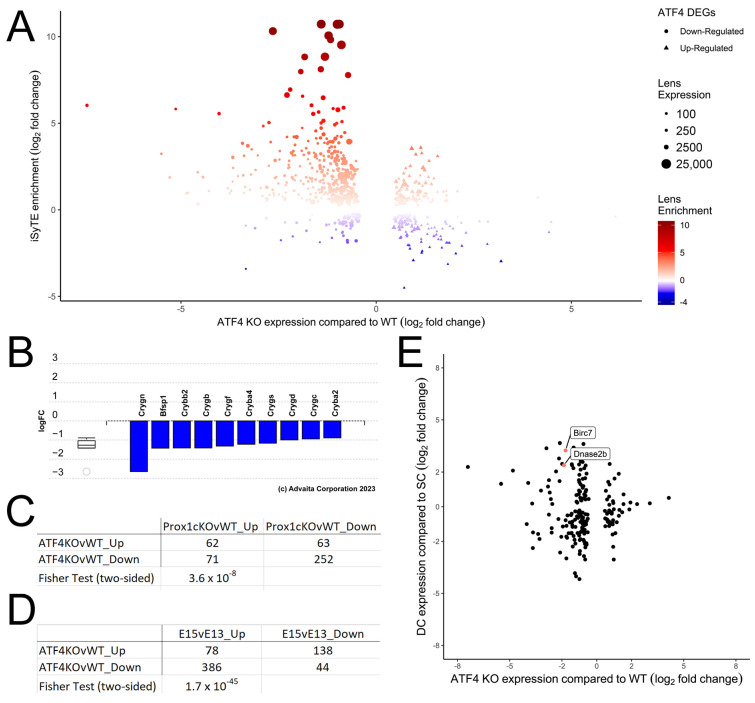
ATF4 likely regulates a portion of the lens-preferred transcriptome including markers of late fiber cell differentiation. (**A**) iSyTE enrichment analysis of *Atf4* null DEGs demonstrating that genes that are downregulated in the *Atf4* null lens are disproportionately over-represented in those exhibiting lens-preferred expression (**B**). Advaita iPathway guide analysis shows that genes differentially expressed in the *Atf4* null lens are over-represented in lens structural proteins. (**C**) Comparison between the DEGs observed in the *Atf4* null lenses with DEGs found in the *Prox1* null lens revealed a significant set of genes whose expression is downregulated in both mutants. (**D**) Comparison between the DEGs detected in the E16.5 *Atf4* null lens with those that change expression between E13.5 and E15.5 in the wildtype lens found that many genes that normally upregulate as embryonic lens development proceeds fail to do so in *Atf4* mutants. (**E**) Comparison between the Atf4 null DEG list with genes whose expression changes as superficial cortical fibers (SC) differentiate into deep cortical fibers (DC) revealed that some known markers of lens fiber cell terminal differentiation such as *Birc7* and *Dnase2b* are downregulated in the E16.5 *Atf4* null lens.

Inspection of the differentially expressed genes (DEGs) in the *Atf4* null lens revealed a number of genes with known functions in the ocular lens (Table 2) and comparison of the *Atf4* null DEGs with the iSyTE database, which evaluates genes for their enriched expression in the lens [69,70], revealed that 148 of the *Atf4* null DEGs exhibit lens-enriched expression in the E16.5 mouse lens (*p* ≤ 1.8 × 10^−65^, Figure 4A, Supplemental Table S2). Notably, 140 of these lens-enriched genes were downregulated in the *Atf4* null lens, and iPathway guide analysis [71] revealed that the *Atf4* null DEGs were significantly over-represented in lens structural constituents (Figure 4B, *p* ≤ 5.8 × 10^−4^). Comparison of the E16.5 *Atf4* null DEGs with DEGs detected in lenses lacking the transcription factor Prox1, which is a key regulator of lens fiber cell differentiation [12], revealed that over 250 genes were downregulated in both mutants (Figure 4C, Table 1, Supplemental Table S3; *p* ≤ 3.6 × 10^−8^) suggesting that ATF4, like Prox1, may regulate the expression of genes important for lens function. As the *Atf4* null lenses form normally, then disintegrate in late embryonic lens development, we then compared the *Atf4* null DEGs with a list of genes that change expression in the mouse lens between E13.5 (right after primary lens fiber cell elongation is complete [12]) and E15.5 (near the onset of fiber cell denucleation and directly before the *Atf4* null phenotype is morphologically apparent [56]). This comparison revealed that over 380 genes that normally upregulate in mid-late lens development fail to do so in the *Atf4* null lens, suggesting that *Atf4* may play a key role in the later stages of lens fiber cell terminal differentiation (Figure 4D, Table 1, Supplemental Table S4). Consistent with this, known late lens fiber cell markers such as *DnaseIIb* (DNase II beta) [72] and *Birc7* [19] were downregulated in *Atf4* null lenses (Figure 4E). Next, we compared the DEGs found in the *Atf4* null lens with those observed in lenses null for *Hsf4*, which encodes a transcription factor important for late lens function [73] (Table 1). Interestingly, while 86 genes were differentially regulated in both *Atf4* and *Hsf4* null lenses (*p* ≤ 1 × 10^−23^), there was not a significant correlation between the direction of their differential regulation (*p* = 0.31, not shown). Overall, these data show that ATF4 is an important player in the gene regulatory network (GRN) controlling the latter stages of embryonic lens development even though it appears to not be directly regulated by other known lens GRN components.

### 3.6. The Atf4 Null Lens Has Reduced mRNA Levels of Many Genes Regulating Amino Acid Metabolism and Reduced Free Amino Acid Pools

**Figure 5:**
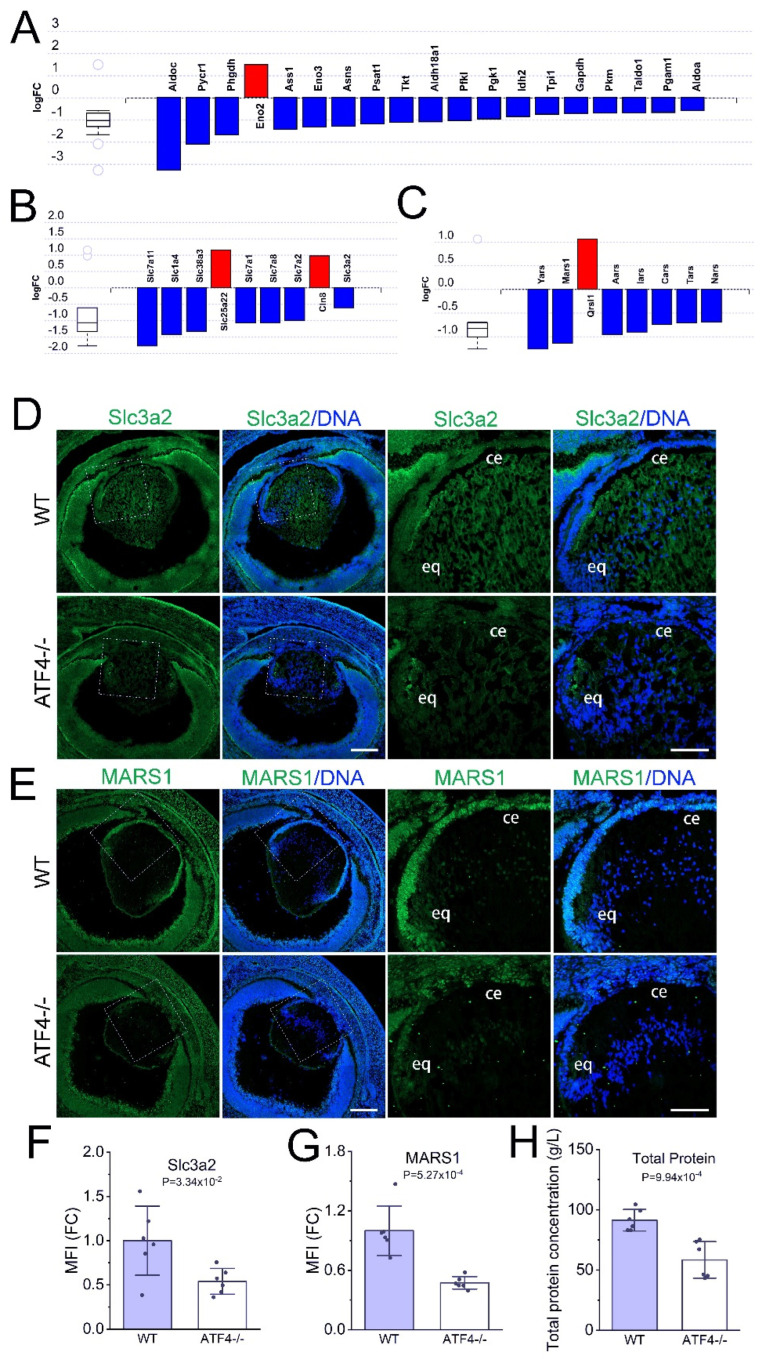
The mRNA levels for many genes encoding regulators of amino acid transport, synthesis, and t-RNA charging are reduced in the *Atf4* null lens. (**A**) Advaita iPathway guide analysis shows that *Atf4* null DEGs are over-represented in mRNAs encoding regulators of amino acid synthesis. (**B**) Several mRNAs encoding amino acids transporters are differentially expressed in the *Atf4* null lens. (**C**) The levels of several mRNAs encoding enzymes responsible for t-RNA charging are reduced in *Atf4* null lenses. (**D**) Immunostaining of E16.5 wildtype and *Atf4* null lens for the amino acid transporter encoded by the *Slc3a2* gene (green) counterstained with Draq5 to detect DNA (blue). Images on the left were generated from a 3 × 3 tile scan with a 20× objective (scale bar = 200 μm), and the boxed region is shown in a zoomed-in view on the right (scale bar = 100 μm). (**E**) Immunostaining of E16.5 wildtype and *Atf4* null lens for the t-RNA charging enzyme Mars1 (green) counterstained with Draq5 to detect DNA (blue). Images on the left were generated from a 3 × 3 tile scan with a 20× objective (scale bar = 200 μm), and the boxed region is shown in a zoomed-in view on the right (scale bar = 100 μm). *Abbreviations:* ce, central epithelium; eq, equator. (**F**) Quantification of the mean fluorescent staining intensity of SLC3A2 immunostaining in the E16.5 wildtype (*n* = 6) and *Atf4* null (*n* = 6) lens. (**G**) Quantification of the mean fluorescent staining intensity of MARS1 immunostaining in the E16.5 wildtype (*n* = 6) and *Atf4* null (*n* = 6) lens. (**H**) Quantification of total soluble protein extractable from the E16.5 wildtype (*n* = 6) and *Atf4* null (*n* = 6) lens expressed as the concentration of protein per lens volume in order to normalize these values to account for the smaller size of the *Atf4* null lens.

iPathway guide impact analysis [71] of the *Atf4* null gene list revealed that the DEGs are enriched in genes regulating amino acid biosynthesis (*p* ≤ 7.8 × 10^−6^; Figure 5A). There is also an enrichment in genes mapping to the KEGG pathway amino acid metabolism (*p* ≤ 0.02, not shown), and these included genes known to be involved in amino acid transport by cells (Figure 5B) and tRNA charging (Figure 5C). Notably, ATF4 binding to genes encoding nutrient transporters and amino-acyl tRNA synthases has been described previously in the literature [74,75], while mining of Chip atlas [76] for the ATF4-binding sites found in the mouse genome revealed that 148 of the differentially expressed genes in the *Atf4* null lens, including nutrient transporters and amino-acyl t-RNA synthases, bind ATF4 within 1 kB of their transcriptional start site in mouse cells (see Supplemental Table S5). This suggests that ATF4 is a direct regulator of genes important for nutrient transport in the avascular lens.

**Figure 6:**
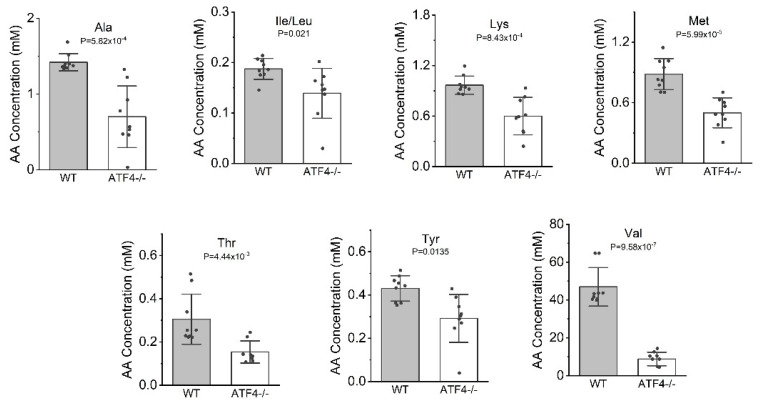
The *Atf4* null lenses have reduced free amino acid pools. Mass spectrometry analysis of free amino acid levels in E16.5 wildtype (*n* = 9) and *Atf4* null (*n* = 9) lenses revealed significant reductions in 7 different amino acids when considered either on the basis of concentration in the lens (normalized to lens volume), while 12 amino acids were found to have significantly lower absolute amounts per lens (Supplemental Figure S3).

Immunostaining for SLC3A2, an amino acid transporter whose mRNA is abundant in the wildtype lens (74 FPKM) but 1.5-fold downregulated in the *Atf4* null lens, revealed that it is broadly distributed in both lens epithelial and fiber cells of the wildtype lens while both expression domains are downregulated in the E16.5 *Atf4* null lens (Figure 5D,F). Consistent with the reduced expression of genes that regulate cellular amino acid concentration, LC-MS analysis of 16 different amino acids found that *Atf4* null lenses have significantly decreased levels of free Ala, Arg, Glu, Gly, Ile/Leu, Lys, Met, Phe, Pro, Thr, Tyr, and Val (Figure 6). Immunostaining for MARS1, which regulates methionine charging to its cognate tRNA [77], revealed that its expression is epithelial-preferred in the wildtype lens, while its levels were decreased in E16.5 *Atf4* null lenses (Figure 5E,G) consistent with the observed 2-fold decrease in its mRNA levels. As expected from these changes in gene expression, *Atf4* null lenses exhibited a 60% reduction in extractable protein compared to wildtype controls (Figure 5H). These data further support the idea that *Atf4* is important for regulating amino acid and protein metabolism in the lens.

### 3.7. Aft4 Null Lenses Have Reduced Levels of Several Glutathione Pathway Genes and Free Glutathione

**Figure 7:**
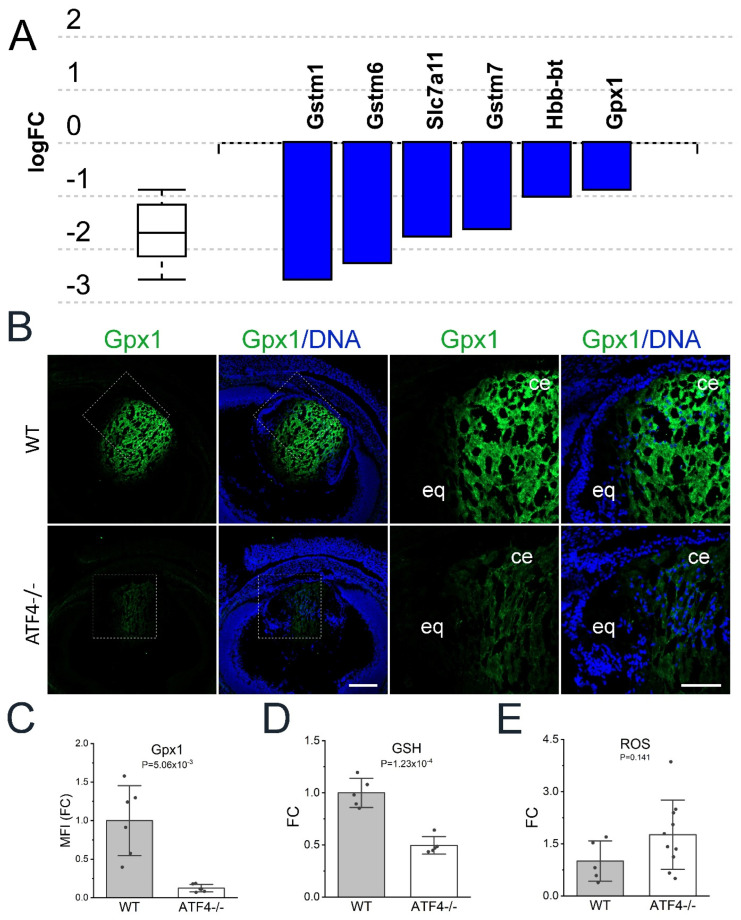
*Aft4* null lenses exhibit reduced mRNA levels of several genes encoding regulators of the glutathione pathway as well as free glutathione. (**A**) Advaita iPathway guide analysis of the DEGs found in the *Atf4* null lens revealed that multiple genes known to regulate glutathione metabolism are downregulated following *Atf4* deletion. (**B**) Immunostaining of E16.5 wildtype and *Atf4* null lens for glutathione peroxidase 1 (Gpx1, green) counterstained with Draq5 to detect DNA (blue). Images on the left were generated from a 3 × 3 tile scan with a 20× objective (scale bar = 200 μm), and the boxed region is shown in a zoomed-in view on the right (scale bar = 100 μm). *Abbreviations:* ce, central epithelium; eq, equator. (**C**) Quantification of the mean fluorescent staining intensity of Gpx1 immunostaining in the E16.5 wildtype (*n* = 6) and *Atf4* null (*n* = 6) lens based on fold change (FC) from mean levels found in the wildtype (WT) lens. (**D**) Quantification of free glutathione (GSH) levels in the E16.5 wildtype (*n* = 5) and *Atf4* null (*n* = 5) lens using the GSH Glo glutathione assay expressed as the fold change (FC) from mean levels found in the wildtype (WT) lens. (**E**) Quantification of reactive oxygen species (ROS) levels in living E16.5 lenses by incubating E16.5 wildtype (*n* = 5) and *Atf4* null (*n* = 10) lenses with non-fluorescent dehydrorhodamine 123 following measurement of ROS-driven production of fluorescent dehydrorhodamine 123 with a plate reader. These data are expressed as fold change (FC) from the mean levels found in the wildtype (WT) lens.

Lenses not only require abundant pools of free amino acids to produce the high concentrations of protein needed for their high refractive index but also require high concentrations of the tripeptide glutathione as it is critical to protect long-lived lens proteins from oxidative damage [78]. Notably, iPathway guide analysis revealed that the mRNA encoding several proteins important for glutathione metabolism (Figure 7A; *p* ≤ 0.003) were downregulated in the *Atf4* null lenses. Of these, glutathione peroxidase 1 (*Gpx1*), an enzyme important for long-term lens transparency that utilizes glutathione to detoxify hydrogen peroxide [79], is abundantly expressed at the mRNA level (175FPKM) in the wildtype embryonic lens, and this decreases 46% in the E16.5 *Atf4* null lens (94 FPKM). Immunolocalization of Gpx1 revealed that this protein is predominately found in the lens core that is predominately comprised of late differentiating fiber cells at this time point, while these levels were significantly reduced in the *Atf4* null lens (Figure 7B,C). We then investigated total glutathione (GSH) levels in E16.5 lenses and found that the *Atf4* null lens has significantly lower levels of GSH than wildtype (Figure 7D). As GSH is a major scavenger of reactive oxygen in the lens [78], we then assayed both wild type and *Atf4* null lenses for ROS (Figure 7E). While some individual *Atf4* null lenses were found to have elevated levels of ROS, as a population, these differences were not statistically significant.

### 3.8. Atf4 Null Lenses May Have Alterations in Sugar Metabolism Compared to Wildtype

**Figure 8:**
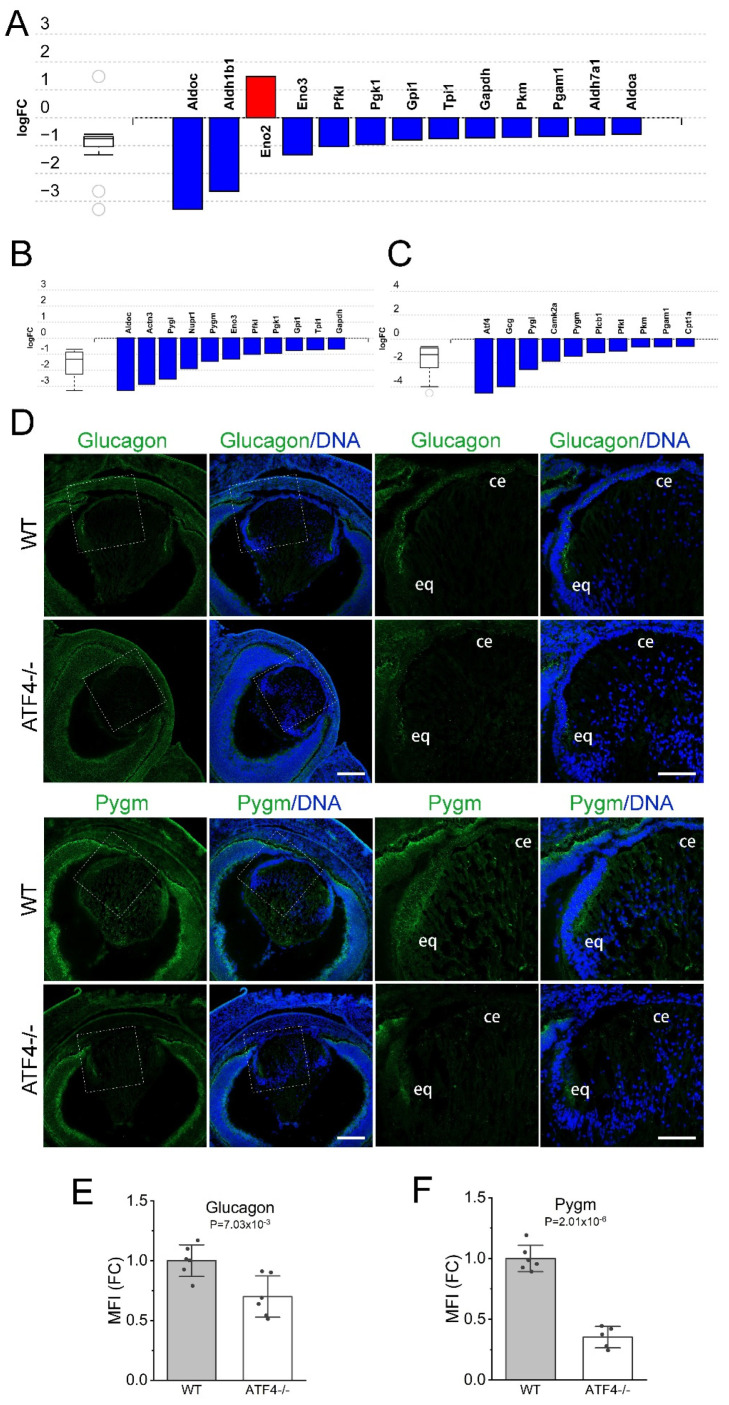
*Atf4* null lenses may have altered sugar metabolism compared to wild type. (**A**) Advaita iPathway guide analysis shows that *Atf4* null DEGs are over-represented in mRNAs encoding regulators of glycolysis/gluconeogenesis. (**B**) Several mRNAs encoding enzymes that function in carbohydrate catabolism are differentially expressed in the *Atf4* null lens. (**C**) The levels of several mRNAs encoding proteins participating in the glucagon pathway are reduced in *Atf4* null lenses. (**D**) Immunostaining of E16.5 wildtype and *Atf4* null lens for glucagon (green) that is encoded by the *Gcg* gene and glycogen phosphorylase (muscle form, green) encoded by *Pygm* counterstained with Draq5 to detect DNA (blue). Images on the left were generated from a 3 × 3 tile scan with a 20× objective (scale bar = 200 μm), and the boxed region is shown in a zoomed-in view on the right (scale bar = 100 μm). *Abbreviations:* ce, central epithelium; eq, equator. (**E**) Quantification of the mean fluorescent staining intensity of glucagon immunostaining in the E16.5 wildtype (*n* = 6) and *Atf4* null (*n* = 6) lens. (**F**) Quantification of the mean fluorescent staining intensity of PYGM immunostaining in the E16.5 wildtype (*n* = 6) and *Atf4* null (*n* = 6) lens.

iPathway guide analysis also predicted that glycolysis/gluconeogenesis pathways as the second most significantly impacted in the *Atf4* null lens (*p* = 2.2 × 10^−5^; Figure 8A) with many of these involved in carbohydrate catabolism (*p* = 0.007; Figure 8B), while numerous genes mapping to the glucagon signaling pathway that includes *Atf4* itself (*p* = 0.003) were downregulated as well (Figure 8C). Consistent with its 16-fold downregulation in mRNA level, immunolocalization of glucagon (Gcg), a peptide hormone that regulates cellular glucose levels [80], revealed that its expression is predominately found in the lens epithelium of the E16.5 wildtype lens, while its levels were reduced in the *Atf4* null lens (Figure 8D,E). Immunostaining of PYGM/Myophosphorylase, an enzyme involved in glycogenolysis [81], revealed that it is expressed in all lens cells at E16.5 although it was more abundant in the lens epithelium, while its expression was significantly decreased in the *Atf4* null lens (Figure 8D,F) consistent with the observed 2.8-fold downregulation of its mRNA levels in the mutant lens.

### 3.9. Only Portions of the Autophagy Pathway Are Induced in the Atf4 Null Lens

The reduced expression of genes involved in nutrient transport and utilization with concomitant reductions in free amino acid levels suggest that the E16.5 *Atf4* null lens is experiencing nutrient deprivation, a situation that would be expected to trigger autophagy via the eIF2α/ATF4 pathway [25]. Since ATF4 is not available in the *Atf4* null lens to drive that function, it is not unexpected that iPathway guide analysis of pathways impacted by *Atf4* deletion only found that 8/132 genes of the pathway “animal autophagy” were affected, and this relationship was not statistically significant (*p* = 0.66). However, P62 (encoded by the *Sqstm1* gene), a common marker of autophagy, was 2.9-fold upregulated in the *Atf4* null lens. This may reflect the 9-fold upregulation of *Ddit3* mRNA in the *Atf4* lens as this gene encodes CHOP, a transcription factor that is a direct transcriptional regulator of *Sqstm1* (p62) gene expression [25]. As expected, immunolocalization revealed little to no detectable P62 staining in the wildtype lens; however, we found that P62 protein levels were also significantly upregulated in the E16.5 *Atf4* null lens, particularly in the transition zone (Figure 2A,C). As the impairment of autophagy results in elevated steady-state P62 protein levels as the protein is degraded during the autophagy process [82], it is possible that this reflects either inefficient autophagy due to the lack of ATF4-mediated induction of autophagy genes or perhaps induction of P62 levels due to other cellular stress responses [83]. As autophagy also requires cap-independent translation via eIF2α phosphorylation, we also evaluated eIF2α levels and activation in the *Atf4* null lens. RNAseq analysis of EIF2S1 expression (the gene that encodes eIF2α) revealed that the wildtype and *Atf4* null lens express similar levels of its mRNA (27 vs. 29 FPKM; Supplemental Table S1). In contrast, immunolocalization of eIF2α protein found that the epithelial cells of the transition zone had modest, but significantly upregulated, levels in the *Atf4* null lens (Figure 2B,D). However, the levels of eIF2α phosphorylation on Ser51, which regulates its function in translational initiation, were not significantly changed in *Atf4* null lenses (Figure 2B,E).

## 4. Discussion

The lens is a unique cellular structure that has numerous structural and biochemical specializations that mediate its transparency and the high refractive index necessary for its function [1]. While our understanding of the key cell signaling cascades and transcription factors that regulate the cell fate decisions driving its early development from the head ectoderm is derived from intensive study over the past 30 years, much less is known about the regulation of key events driving the maturation of the lens into its final functional form. Notably, while the transcription factor ATF4 was first reported to play a critical role in late embryonic lens cell survival over 25 years ago [30], neither its position in the gene regulatory network driving lens development nor its function in the lens had been previously explored. This report seeks to close those knowledge gaps.

### 4.1. ATF4 as a Component of the Gene Regulatory Network (GRN) Regulating Lens Development

Atf4 mRNA expression has been reported to be epithelial-preferred in E14.5 mouse lenses via in situ hybridization [29]; however, bulk RNAseq profiling indicates that *Atf4* mRNA is approximately 2-fold more abundant in mouse lens fibers than LECs [22,84]. ATF4 protein is detectable in all embryonic lens cells at E11.5, then decreases in lens fibers after E13.5 while remaining high in LECs [48]. Interrogation of previously reported gene expression profiles performed on lenses lacking key lens transcription factors revealed that *Atf4* mRNA was only downregulated 1.5-fold in *Prox1* null lenses [12], while *Atf4* expression was unaltered in lenses homozygous for *Hsf4* [73], homozygous for *Sip1* [56], or heterozygous for *Pax6* mutations [85]. In contrast, *Atf4* mRNA levels are induced 2.5-fold in 56-day-old *Klf4* null lenses, although they are unchanged in E16.5 *Klf4* nulls [86], the timepoint where the *Atf4* null phenotype manifests. Inspection of the DEG list from E16.5 *Atf4* null lenses revealed that the expression levels of genes encoding the vast majority of transcription factors known to regulate lens development including *Pax6*, *Prox1*, *Pitx3*, *cMaf*, *Ap2α*, *Sox2*, *Meis1/2*, and *Hsf4* were unaffected demonstrating that ATF4 is likely not upstream of these genes in the lens GRN. A notable exception to this observation was that the mRNA encoding the transcription factor *FoxE3*, a regulator of both early lens development and later lens epithelial phenotype [16,17], was 4.7-fold downregulated in the *Atf4* null lens. While this could indicate that ATF4 regulates the *FoxE3* gene directly, the late onset of the *Atf4* null lens phenotype compared to that of *FoxE3* mutants suggests that the loss of *FoxE3* expression is secondary to the apoptotic loss of the lens epithelium/induction of alpha-smooth muscle actin expression in *Atf4* nulls. Overall, these observations and the grossly normal initial morphogenesis of the *Atf4* null lens suggest that this gene is regulated independently of the GRN controlling early lens development.

### 4.2. ATF4 Appears to Not Regulate Developmental UPR in the Lens

ATF4 is a known regulator of the unfolded protein response (UPR), a molecular mechanism that fine-tunes the folding capacity of the endoplasmic reticulum (ER) to the flux of proteins transiting the secretory pathway in normal cells [87]. In cells unable to achieve this balance due to the presence of “unfoldable” mutant proteins or other failures of ER quality control, ATF4 participates with the other arms of the UPR pathway to trigger apoptotic pathways [26]. In the lens, modest UPR pathway activation was detected during normal lens development [48], while the presence of high levels of chronically unfoldable protein leads to activation of all three UPR arms, including elevated levels of ATF4 protein, elevation of UPR markers such a BIP, and low levels of apoptosis [49]. In contrast, we show here that while lenses lacking ATF4 undergo apoptosis, bioinformatic predictions did not reveal UPR as an enriched pathway in *Atf4* null lenses, and BIP (*HspA5*) mRNA levels are not altered even though its protein levels are modestly attenuated. Unexpectedly, *Atf4* null lenses exhibit a 9-fold elevation in *Ddit3*/*Chop* mRNA levels even though ATF4 is a known activator of *Ddit3* transcription during the UPR [52]. While elevations in *Ddit3* expression may explain why *Atf4* null lenses undergo apoptosis as it is considered a pro-apoptotic transcription factor [88], CHOP protein levels appear unaltered.

### 4.3. ATF4 Likely Regulates Genes Involved in Amino Acid Transport and Glutathione Pathways in Lens

ATF4 is selectively translated in cancer cells in response to amino acid starvation during the integrated stress response (ISR) via the action of the protein kinase GCN2 (encoded by the *Eif2ak4* gene) that senses low cellular amino acid levels. The newly produced ATF4 then transcriptionally activates numerous genes involved in amino acid homeostasis including those involved in amino acid synthesis and transport [27,89]. ATF4 production can also be induced by the activation of the mTORC1 complex that promotes both protein and glutathione synthesis in conditions conducive to cell growth although mTORC1-induced ATF4 only regulates a subset of the genes compared to ISR-induced ATF4 expression, predominately those involved in amino acid metabolism and aminoacyl tRNA synthesis [90].

Here we found that at least one major ATF4 function during lens development is the regulation of cellular amino acid levels and protein synthesis as *Atf4* null lenses exhibit reduced levels of mRNAs encoding numerous proteins involved in amino acid synthesis and transport as well as critical regulators of t-RNA charging and glutathione metabolism. Notably, a comparison of the *Atf4* null lens DEGs with the Chip-Atlas database [76,91] revealed that many of the DEGs in the *Atf4* null lens are direct ATF4 target genes in other cellular contexts. These defects in gene expression likely explain the observation that *Atf4* null lenses have significant reductions in free amino acid, glutathione, and total protein content.

### 4.4. ATF4 Likely Functions to Metabolically Adapt the Lens to Its Avascular Environment

**Figure 9:**
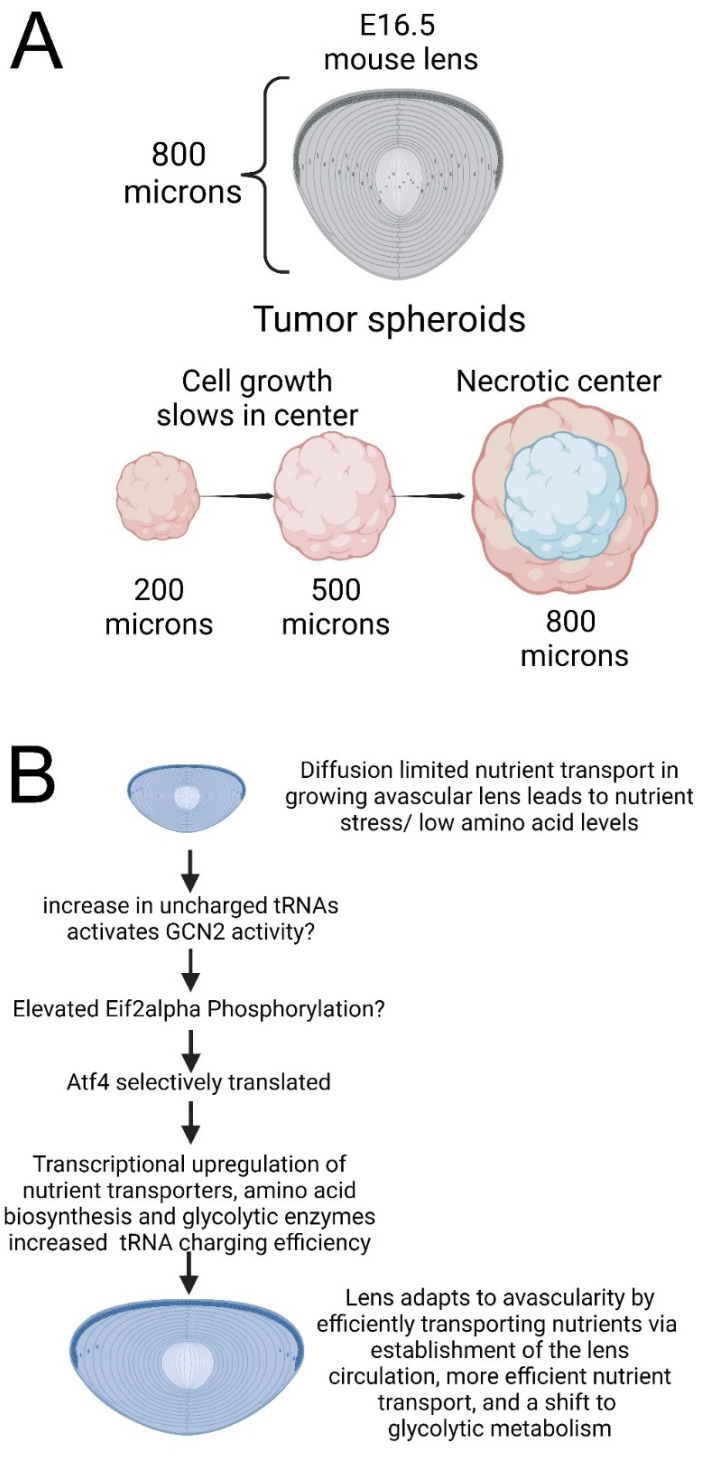
*Atf4* likely functions to metabolically adapt the lens to its avascular environment. (**A**) The normal embryonic mouse lens can grow to 800 microns in diameter by E16.5 without an invasive vasculature, while the center of avascular tumor organoids that have reached this size is typically apoptotic/necrotic due to nutrient deprivation [106,107]. (**B**) Increasing the diameter of the embryonic lens along with the regression of the lens-associated tunica vasculosa lentis leads to nutrient stress/low amino acid levels, likely resulting in increases in uncharged tRNAs triggering the activation of GCN2 that results in selective translation of ATF4, so it can mediate transcriptional upregulation of nutrient transporters as well as amino acid biosynthesis and glycolytic enzymes, eventually increasing nutrient supply and tRNA charging efficiency. In conclusion, the lens adapts to avascularity by efficiently transporting nutrients via the establishment of the lens circulation, more efficient nutrient transport, and an ability to make ATP in a low-oxygen environment. Figure produced with Biorender.

The ocular lens must remain avascular throughout life as the presence of blood/blood vessels in the optical axis would prevent light transmission to the retina although the lenses of most species are much larger than the maximum diameter that most cellular organoids/tissues/cancers can reach without a blood supply [92]. Notably, E16.5, the stage where the *Atf4* null mouse lens becomes phenotypically abnormal, is approximately 800 microns in diameter, while the center of avascular tumor organoids that have reached this size is typically apoptotic/necrotic due to nutrient deprivation and tissue hypoxia [93] (Figure 9A). The lens can function without a vasculature because it has evolved an intrinsic circulatory system in which sodium/potassium ATPase activity in the lens epithelium provides a motive force to move water and small nutrients/metabolites through the pericellular space while high levels of gap junctional coupling between lens fiber cells allow movement of small molecules within the fiber cells themselves [8].

However, even with an active lens circulation, it would be expected that the increasing diameter of the lens during embryonic development, along with the regression of the lens-associated tunica vasculosa lentis (which has begun by birth in mice) [94], would result in a drop in free amino acid levels and an increase in uncharged tRNAs within lens cells, potentially leading to the activation of GCN2 and subsequent translation of ATF4. This would result in the elevated expression of numerous nutrient transporters and amino acid biosynthesis enzymes while increasing the efficiency of tRNA charging by increasing the synthesis of tRNA synthetases that would collaborate to allow the lens to synthesize the high concentrations of protein needed for its refractive function (Figure 9B). As *Atf4* null lenses show reduced amino acid and protein levels, an increase in the autophagy marker P62, and finally disintegrate by birth via apoptosis, it is likely that ATF4 is critical for lenses to effectively adapt to their low-nutrient environment. Future studies are needed to determine whether *Atf4* is regulated by GCN2, mTORC1, or a combination of these pathways to increase the efficiency of nutrient transport in the lens as it outgrows the ability to bring in nutrients via diffusion.

Most ATP production in the adult lens is produced by glycolysis, likely due to the intrinsic hypoxia of the lens environment [95]. While genes mapping to the gene ontology terms related to “hypoxia” are not over-represented among the *Atf4* null DEGs, numerous genes encoding proteins important for glycolysis are expressed at lower levels in *Atf4* null lenses than littermate controls, a result consistent with prior reports that ATF4 mediates stress-induced shifts towards glycolytic metabolism in other cell types [96,97]. Notably, HIF1a, the canonical transcription factor mediating survival of hypoxic cells, is also abundant in the lens and necessary for postnatal lens survival and growth in mice [98,99] and lens fiber cell denucleation in chickens [100]. Both ATF4 and HIF1a pathways are often activated during cellular stress responses [101], and like HIF1a, ATF4 levels are oxygen-sensitive due to interactions with the PHD3 oxygen sensor [102]. However, the regulatory relationship between ATF4 and HIF1a is likely complex and may be cell-type-dependent. For instance, HIF1a blocks ATF4 expression in early cardiomyocytes even in the presence of cellular stress by binding to the *Atf4* promoter [103], while ATF4 and HIF1a proteins may physically interact in adult cardiomyocytes potentially enhancing hypoxia-induced cell death [104].

Notably, *Hif1a* mRNA levels are not altered in *Atf4* null lenses and treatment of chicken lens epithelial cells with an activator of HIF1α pathways induced the expression of numerous genes associated with glycolysis without altering *Atf4* mRNA levels [105]. This suggests that HIF1α and ATF4 may function independently to establish glycolytic metabolism in the lens to sustain cellular ATP levels as the growing lens becomes increasingly hypoxic. Additional investigations will be required to determine whether ATF4 function in the lens is primarily related to establishing the needed amounts of nutrient transporter and glycolytic enzyme expression in the avascular lens or also helps the lens adapt to a low-oxygen environment in collaboration with Hif1α.

## 5. Conclusions and Outstanding Questions

ATF4 is a transcription factor important for lens biology that likely acts independently of the classic lens development GRN to mediate the adaption of the lens to the nutrient deprivation resulting from its avascularity. It is currently unknown how *Atf4* transcript levels are regulated in the developing lens, the precise time point in lens development when ATF4 function becomes necessary, the key mechanisms that fine-tune its function to drive appropriate lens amino acid levels, or whether/how it interfaces with HIF1a during adaption of the lens to chronic tissue hypoxia. Notably, *Atf4* mRNA levels also upregulate sharply in lens epithelial cells by 6 h after lens injury and remain elevated for several days, while *Hif1a* mRNA levels decrease in injured LECs over this same time frame [22,61,108,109]. As ATF4 is best known as a mediator of cellular responses to acute stress in other tissues [26], the lens may be an ideal system to investigate how the diverse functions of Atf4 are differentially regulated during both normal tissue development and as a consequence of injury responses.

## Figures and Tables

**Table 1:** Accession numbers used for cross-experiment comparisons in this study.

Name of Experiment	GEO Accession	Experiment Type	Library Source
Lens Superficial Cortex vs. Lens Epithelial Cells	GSE205379	Microarray	Transcriptomic
Lens Deep Cortex vs. Lens Superficial Cortex	GSE205379	Microarray	Transcriptomic
Prox1 Knockout vs. Wildtype	GSE69940	RNA-Seq	Transcriptomic
HSF4 Knockout vs. Wildtype	GSE22362	Microarray	Transcriptomic
Whole Lens E15.5 vs. Whole Lens E13.5	E15.5 (GSE49949) E13.5 (GSE69940)	RNA-Seq	Transcriptomic
ATF4 Knockout vs. Wildtype	GSE35681	ChIP-Seq	Genomic

**Table 2:** Genes with known lens expression/function differentially expressed in the E16.5 *Atf4* null lens.

Symbol	Description	Citation	Fold Change	FDR	Wildtype FPKM	*Atf4* KO FPKM
*Foxe3*	Forkhead box E3	[16]	−4.7	1.56 × 10^−4^	140	30
*Dnase2b*	Deoxyribonuclease II beta	[62]	−3.7	2.72 × 10^−8^	31	8.6
*Birc7*	Baculoviral IAP repeat-containing 7	[19]	−3.5	6.20 × 10^−5^	18	5.3
*Bfsp1*	Beaded filament structural protein 1	[63]	−2.7	6.06 × 10^−5^	2924	1094
*Crybb2*	Crystallin, beta B2	[64]	−2.7	9.45 × 10^−3^	23	8.8
*Crygb*	Crystallin, gamma B	[65]	−2.7	6.84 × 10^−5^	5582	2103
*Crygf*	Crystallin, gamma F	[65]	−2.5	8.17 × 10^−4^	7129	2869
*Cryba4*	Crystallin, beta A4	[65]	−2.3	2.72 × 10^−4^	4983	2138
*Crygs*	Crystallin, gamma S	[65]	−2.3	1.71 × 10^−3^	280	125
*Hopx*	HOP homeobox	[66]	−2.1	3.59 × 10^−2^	12	5.8
*Crygd*	Crystallin, gamma D	[65]	−2.0	9.70 × 10^−3^	5561	2783
*Caprin2*	Caprin family member 2	[67]	−2.0	6.24 × 10^−3^	100	51
*Crygc*	Crystallin, gamma C	[65]	−1.9	1.35 × 10^−2^	2881	1496
*Cryba2*	Crystallin, beta A2	[65]	−1.9	1.74 × 10^−2^	3397	1831
*Hmgn3*	High mobility group nucleosomal binding domain 3	[68]	−1.8	1.12 × 10^−2^	137	78.50

## Data Availability

RNAseq data produced in this study have been deposited in the Gene Expression Omnibus under accession number GSE206760.

## Supplementary Materials

The following supporting information can be downloaded at https://www.mdpi.com/article/10.3390/cells12222636/s1, Figure S1: Hematoxylin and eosin staining of the E14.5 wildtype and *Atf4* null lens. Figure S2: Expression of BiP, CHOP, and XBP1 protein in the E16.5 *Atf4* null lens. Figure S3: *Atf4* null lenses differentially regulate many genes and lack *Atf4* expression. Figure S4: The *Atf4* null lenses have reduced free amino acid pools when measured on a per lens basis; Table S1: Genes that are differentially expressed in the E16.5 *Atf4* null lens. Table S2: Genes that are differentially expressed in the E16.5 *Atf4* null lens which are predicted to be lens-enriched by iSyTE. Table S3: Genes that are differentially expressed in the E16.5 *Atf4* null lens that are also differentially expressed in the E13.5 *Prox1* null lens. Table S4: Genes that are differentially expressed in the E16.5 *Atf4* null lens that normally change expression in the lens between E13.5 and E15.5. Table S5: Genes that are differentially expressed in the E16.5 *Atf4* null lens that have experimentally determined ATF4 binding sites in their 5’ flanking sequence. Table S6: Detailed immunostaining conditions for the antibodies used in this study.
