# Supplementary figures and images for: ATF4 May Be Essential for Adaption of the Ocular Lens to Its Avascular Environment
Source: Cells. 2023 Nov 16;12(22):2636. doi: 10.3390/cells12222636 (PMC10670291; doi:10.3390/cells12222636)

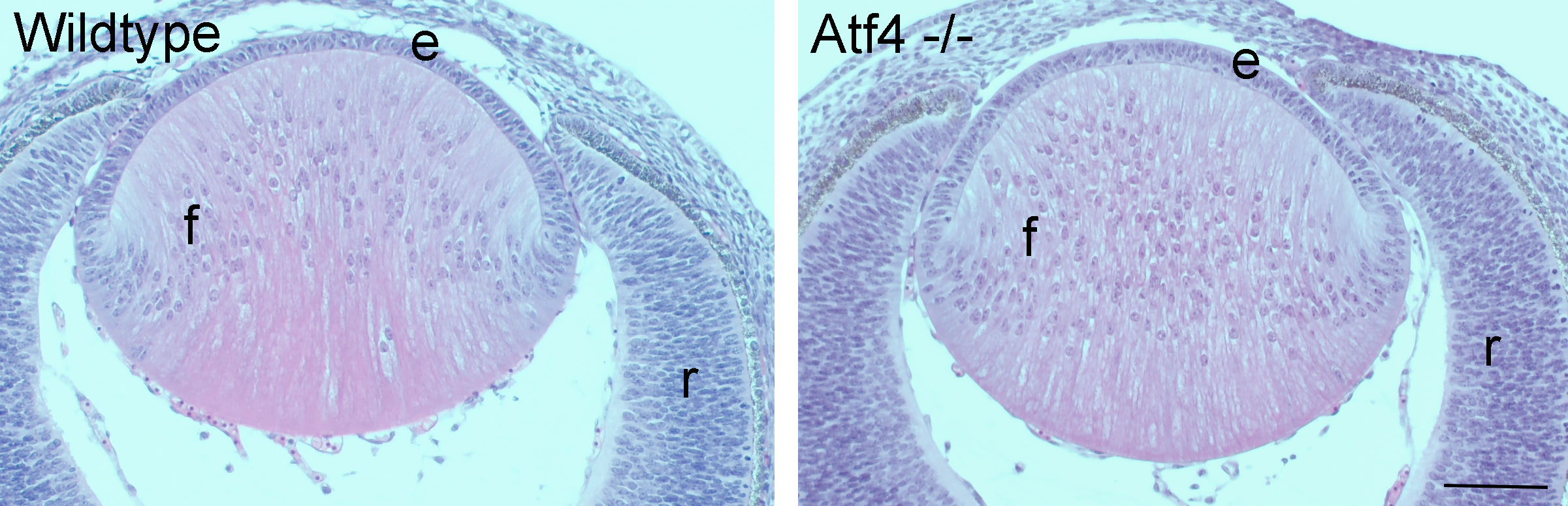

Supplement: Supplementary file 1 [file cells-12-02636-s001.zip › supplemental figure S1 9_23_ 14 day histologycropped.tif]

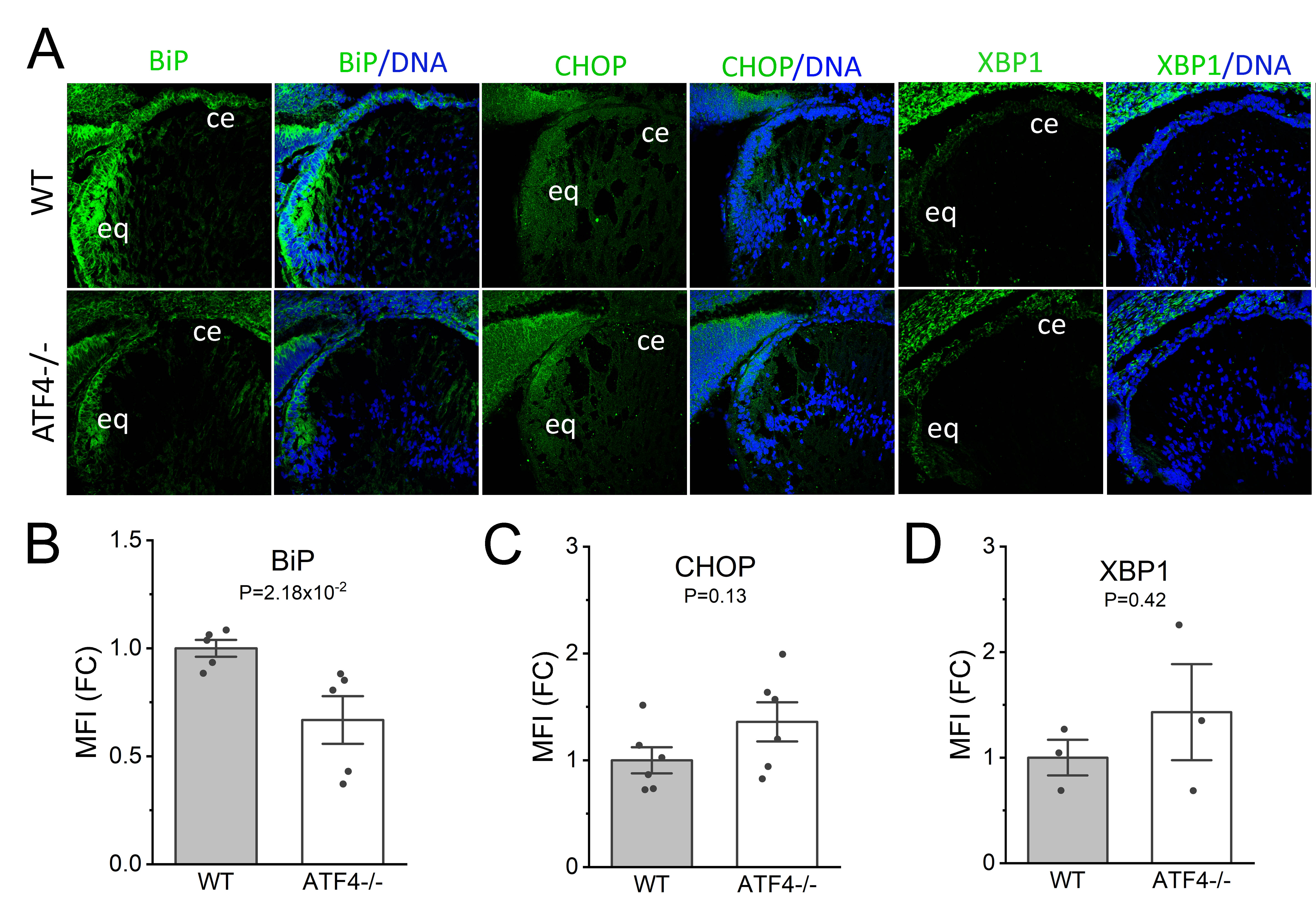

Supplement: Supplementary file 1 [file cells-12-02636-s001.zip › Supplemental Figure S2revised 2_23 copy.jpg]

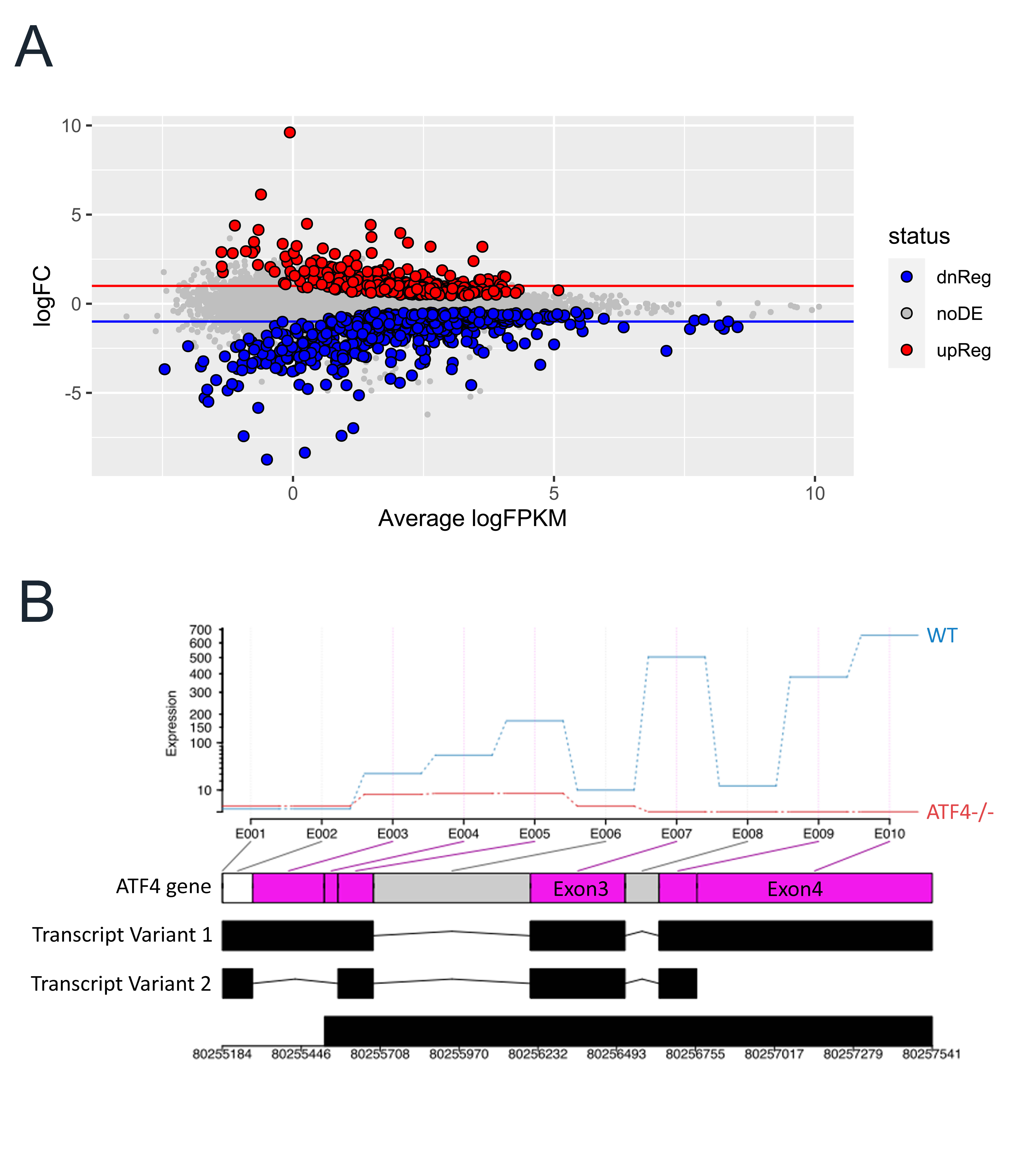

Supplement: Supplementary file 1 [file cells-12-02636-s001.zip › Supplemental Figure S3revised 10_22 copy.jpg]

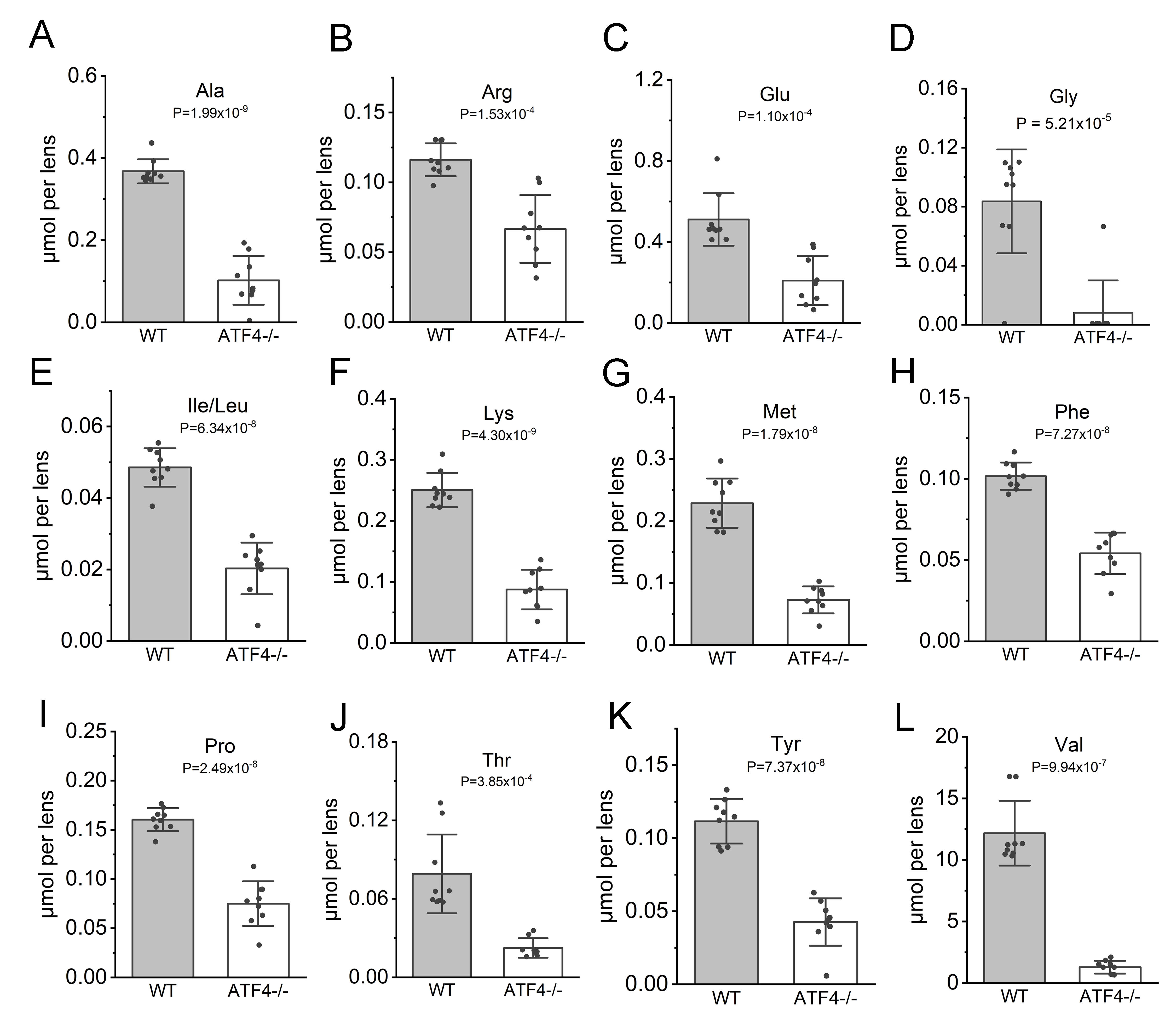

Supplement: Supplementary file 1 [file cells-12-02636-s001.zip › Supplemental figure S4 (AA micromol per lens)revised 3_23 copy.jpg]
